# Cross-Category Screening of Food Samples for Amyloid-β42 Aggregation-Inhibitory Activity Using a Microliter-Scale High-Throughput Screening System with Quantum-Dot-Labeled Aβ

**DOI:** 10.3390/foods15122108

**Published:** 2026-06-11

**Authors:** Kota Nakamura, Manae Kawamura-Yamagishi, Masahiro Kuragano, Koji Uwai, Kiyotaka Tokuraku

**Affiliations:** Graduate School of Engineering, Muroran Institute of Technology, 27-1 Mizumoto-cho, Muroran 050-8585, Hokkaido, Japan; x9pgeavt@gmail.com (K.N.); gano@muroran-it.ac.jp (M.K.); uwai@muroran-it.ac.jp (K.U.)

**Keywords:** Alzheimer’s disease, amyloid-β42, Aβ aggregation inhibitor, food screening, QDAβ, functional food

## Abstract

Alzheimer’s disease (AD) is associated with the aggregation and deposition of amyloid-β (Aβ), making Aβ aggregation an important target in AD-related research. Food-derived components have attracted attention as potential modulators of Aβ-related processes, but the direct effects of diverse food samples on Aβ42 aggregation remain unclear. Here, we screened 120 food-sample preparations derived from 115 food items for inhibitory activity against Aβ42 aggregation using an automated microliter-scale high-throughput screening system with quantum-dot-labeled Aβ (QDAβ). Among primary screening samples, 34 showed detectable Aβ42 aggregation-inhibitory activity, and 12 were classified as highly active (1/EC_50_ ≥ 10 mL/mg). Within the present screening set, highly active samples were frequently observed among tea-related samples. Black tea, Camembert, Red perilla, and Black soybean were selected as representative hits for further validation. Automated MSHTS images and dose–response data showed concentration-dependent suppression of Aβ42 aggregate formation. These inhibitory effects were further supported by thioflavin T (ThT) assays and transmission electron microscopy, which showed suppression of ThT-positive fibrillar aggregation and reduced fibrillar aggregate formation. In differentiated PC12 cells, selected food samples increased cell viability in Aβ42-treated cells at some concentrations. These findings provide a basis for functional food research and active component analysis of food-derived Aβ42 aggregation modulators.

## 1. Introduction

Alzheimer’s disease (AD) is a neurodegenerative disease characterized by progressive cognitive decline and accounts for approximately 60–70% of dementia cases [1,2]. The number of people living with dementia is increasing worldwide; approximately 57 million people were estimated to have dementia in 2021, with nearly 10 million new cases occurring each year [2]. The global economic cost of dementia reached USD 1.3 trillion in 2019 and is projected to increase to USD 2.8 trillion by 2030 [3]. In addition, the number of people with dementia is estimated to increase from 57.4 million in 2019 to 152.8 million by 2050 [4]. Thus, dementia, including AD, represents a major medical, social, and economic challenge. Pathologically, AD is characterized in part by senile plaque formation involving the accumulation of amyloid-β (Aβ) [5]. Aβ progressively assembles from monomers into oligomers, protofibrils, and mature fibrils, and these aggregation processes are thought to be associated with neurotoxicity and AD pathogenesis [6,7]. In particular, Aβ42 is more aggregation-prone than Aβ40 [8] and has been widely studied as an important target molecule in AD-related aggregation research. Therefore, identifying substances that modulate Aβ42 aggregation and deposition is an important research objective for understanding Aβ-related pathology and exploring candidates for preventive intervention.

Recent clinical application of anti-Aβ antibodies that remove Aβ deposits, such as lecanemab [9] and donanemab [10], has advanced. However, these therapies are mainly intended for patients with early AD and slow the progression of cognitive decline, rather than sufficiently restoring cognitive impairment that has already occurred. Indeed, lecanemab significantly slowed cognitive and functional decline in patients with early AD, but its effect was limited to delaying disease progression [9]. Donanemab has also been reported to slow clinical progression in early symptomatic AD [10,11]. These findings indicate that interventions targeting Aβ deposition can have clinical efficacy, while also highlighting the difficulty of sufficiently reversing disease progression after symptom onset. The accumulation of Aβ and changes in neurodegeneration-related biomarkers associated with AD development are thought to begin decades before cognitive impairment becomes apparent [12,13]. Therefore, it is important to explore preventive approaches that can modulate Aβ-related processes before symptoms become clinically evident.

Foods and food-derived components have attracted broad interest in functional food research for AD prevention because they are consumed on a daily basis. Epidemiological studies have reported associations between dietary patterns, such as the Mediterranean diet, MIND diet, and Japanese dietary pattern, and cognitive decline or dementia risk [14,15,16,17]. In addition, dementia prevention studies based on multidomain lifestyle interventions combining dietary improvement, physical activity, cognitive training, and vascular risk management have been developed internationally. In trials and initiatives such as the FINGER trial, World-Wide FINGERS, and U.S. POINTER, nutrition and dietary habits are positioned as important components of preventive intervention [18,19,20]. Furthermore, rosmarinic acid has been shown to suppress Aβ aggregation in AD model mice [21], and extracts of *Perilla frutescens* var. *acuta* (L.) Britt. (Lamiaceae) have been suggested to suppress Aβ deposition and neuroinflammation in 5XFAD mice [22]. These findings suggest that food-derived components may influence Aβ-related processes in vivo.

On the other hand, studies of dietary patterns and lifestyle interventions cannot readily identify which food samples or food-derived components directly affect Aβ42 aggregation. In addition, foods are complex multicomponent matrices, and their Aβ42 aggregation-inhibitory activity may vary depending on food type, processing conditions, extraction conditions, storage conditions, and other factors. Therefore, as a first step toward functional food research and active component analysis, an in vitro screening system that enables cross-category comparison of diverse food samples under uniform conditions is important.

The amount of Aβ aggregates has commonly been evaluated using Aβ-binding dyes, such as thioflavin T (ThT) and Congo red. However, in dye-binding assays, fluorescence intensity can be affected by coexisting sample-derived components or by inner filter effects caused by inhibitor candidates themselves, which may lead to false-positive results [23]. This represents a major challenge in screening for novel active compounds. To address this issue, we previously developed a microliter-scale high-throughput screening (MSHTS) method [24] based on visualization of Aβ aggregates using QDAβ [25]. In this method, QDAβ is co-aggregated with unlabeled Aβ42, and Aβ42 aggregation-inhibitory activity is evaluated by quantifying the heterogeneity of fluorescence images as the standard deviation of pixel intensity. Furthermore, automated MSHTS combined with an automated dispensing system has enabled comparison of many samples under uniform assay conditions [26]. We have previously applied the MSHTS method to food-, plant-, and natural product-derived samples. For example, in a study of commercial dressings and soy sauce, soy sauce-based dressings and raw soy sauce were reported to show Aβ42 aggregation-inhibitory activity [27]. In *Perilla frutescens* var. *crispa* leaf extracts, cultivation conditions were shown to affect Aβ aggregation-inhibitory activity [28]. In addition, an automated MSHTS approach was used to search for fractions with Aβ aggregation-inhibitory activity in a library of 210 mushroom extracts [29]. These recent method-related applications indicate that MSHTS is a useful primary screening platform for evaluating the Aβ aggregation-inhibitory activity of complex food-, plant-, and natural-product-derived samples.

However, previous studies have mainly focused on specific food groups, plant materials, or extract libraries, and systematic cross-category comparisons of diverse food samples under the same assay conditions remain limited. In addition, food samples are complex matrices, and screening hits require validation using assays that capture different aspects of Aβ42 aggregation and cellular responses. Therefore, cross-category comparison of food samples using the same automated MSHTS platform, followed by stepwise validation of representative hits using independent aggregation assays and a cell-based assay, is important for prioritizing food-derived candidates for functional food research and active component analysis. These points highlight the unmet need for a standardized cross-category screening approach that compares diverse food samples under the same assay conditions and links primary screening hits to downstream validation using independent aggregation assays and a cell-based assay. We hypothesized that cross-category screening under uniform automated MSHTS conditions would identify food-derived candidates with distinct Aβ42 aggregation-inhibitory activity, and that representative hits could be prioritized by stepwise validation using orthogonal aggregation and cell-based assays.

In this study, we used automated MSHTS to compare the Aβ42 aggregation-inhibitory activity of food samples across multiple food categories and to identify food-derived candidates that modulate Aβ42 aggregation. Representative hit foods were then subjected to stepwise validation using the ThT assay, transmission electron microscopy (TEM), and MTT assay with differentiated PC12 cells to examine whether their aggregation-inhibitory effects were supported by independent assays and whether they showed increased cell viability in Aβ42-treated cells. These analyses showed that diverse food samples include candidates capable of suppressing Aβ42 aggregation, some of which also showed increased cell viability in Aβ42-treated cells, providing a basis for further functional and active-component analyses.

## 2. Materials and Methods

### 2.1. Preparation of Food Extract Samples

Food sample selection and preparation were performed with Air Water Inc. (Osaka, Japan); the complete sample list is shown in Table S1. The primary automated MSHTS dataset consisted of 120 food-sample preparations from 115 food items, because some foods were evaluated under different preparation or storage conditions; these condition-specific preparations were treated as separate samples. Food samples were homogenized using a hand mixer (HB865GJP, T-fal, Rumilly, France) in turbo mode until a homogeneous slurry or powder was obtained. The homogenized samples were frozen in liquid nitrogen and stored at −80 °C until freeze-drying using a freeze dryer (FDU-2200, EYELA, Tokyo, Japan). After freeze-drying, 1 g of each lyophilized sample was placed in a 15 mL screw-cap glass tube (No. N-16, Maruemu, Osaka, Japan), mixed with 50% ethanol as the extraction solvent, and shaken at 200 rpm for 15 h using a multi-shaker (MMS-5020, EYELA, Tokyo, Japan). A 50% ethanol solution was used as the extraction solvent because it can extract a broad range of water-soluble to moderately polar food-derived components, including polyphenols and other small molecules, while remaining compatible with the downstream in vitro screening workflow after drying and reconstitution. In addition, in a preliminary comparison of extraction solvents using tea-related samples, 50% ethanol extracts showed high Aβ42 aggregation-inhibitory activity among the tested solvent conditions (Figure S5). Based on these preliminary results and workflow compatibility, 50% ethanol was selected as the standardized extraction solvent for the present screening. The extracts were centrifuged at 1800× *g*, and supernatants were filtered through PTFE membrane filters (TORAST Disc GLCTD-PTFE1322, Shimadzu GLC, Tokyo, Japan). Filtrate aliquots (1 mL) were dried using a centrifugal evaporator (CVE-3000, EYELA, Tokyo, Japan). The dried extracts were dissolved in DMSO at 100 mg/mL, intermittently sonicated for a total of 300 s (5 min) at room temperature without active temperature control to facilitate dissolution of poorly soluble components, vortexed, and stored at −80 °C until use.

### 2.2. Preparation of Soy Sauce Samples

Commercial soy sauce (Tokkyu Honjoyu, Nihon Shoyu Kogyo Co., Ltd., Hokkaido, Japan) and raw soy sauce (salt content, 16.2%; Nihon Shoyu Kogyo Co., Ltd.) were purchased and processed separately from the dried food extracts. Soy sauce samples were centrifuged at 15,000× *g* for 30 min at 4 °C using a centrifuge (MX-307, TOMY Digital Biology Co., Ltd., Tokyo, Japan), and the supernatants were collected after removal of precipitates.

Because soy sauce samples were liquid, their concentrations were initially prepared on a volume/volume basis. For comparison with dried food extracts, the concentrations of soy sauce and raw soy sauce were determined using the Lowry method [30]. The concentrations of undiluted soy sauce and raw soy sauce were calculated as 30.30 mg/mL and 20.89 mg/mL, respectively, and these values were used to convert *v*/*v* concentrations to mg/mL for EC_50_ calculation.

### 2.3. Preparation of Aβ Peptides

Human Aβ42 peptide (4349-v, Peptide Institute, Inc., Osaka, Japan) and Cys-linked Aβ40 peptide (23519, AnaSpec, Fremont, CA, USA) were purchased commercially. Human Aβ42 was dissolved in 1,1,1,3,3,3-hexafluoro-2-propanol (HFIP; 083-04233, FUJIFILM Wako Pure Chemical Corporation, Osaka, Japan) at 1 mg/mL, incubated at room temperature for 1 h, and sonicated for 10 min to monomerize the peptide. The solution was then dried as a thin film in a clean bench. The dried peptide film was dissolved in dimethyl sulfoxide (DMSO; 041-29351, FUJIFILM Wako Pure Chemical Corporation, Osaka, Japan) to prepare 1 or 2.5 mM Aβ42 stock solutions. The solution was mixed by pipetting for 15 min, aliquoted into 1.5 mL tubes (SARSTEDT, Nümbrecht, Germany), and stored at −80 °C until use.

### 2.4. Preparation of QDAβ Nanoprobe

The QDAβ nanoprobe was prepared using QD-PEG-NH_2_ (Qdot™ 605 ITK™ Amino (PEG) quantum dot; Q21501MP, Thermo Fisher Scientific, Waltham, MA, USA) according to our previous reports [24,25,26], with the key steps summarized here. Briefly, the buffer of QD-PEG-NH_2_ was exchanged to PBS using a centrifugal concentrator. The quantum dots were reacted with the heterobifunctional crosslinker Sulfo-EMCS, and excess crosslinker was removed using a desalting column. Cys-linked Aβ40 dissolved in DMSO was then added to the activated quantum dots to conjugate Aβ40 to the QD surface. Unreacted maleimide groups were quenched with 2-mercaptoethanol, and the reaction mixture was further purified by buffer exchange and desalting. The concentration of QDAβ was determined from the absorbance at 350 nm.

### 2.5. MSHTS System

#### 2.5.1. Evaluation of Aβ42 Aggregation-Inhibitory Activity by MSHTS

Aβ42 aggregation-inhibitory activity was evaluated using a microliter-scale high-throughput screening (MSHTS) method with QDAβ as described previously, with minor modifications. Food extract samples were diluted to 2 mg/mL in assay buffer containing 1× PBS, 10% ethanol, and 2% DMSO. Soy sauce samples were prepared separately at 20% in 1× PBS and 10% ethanol. Assay buffer containing 1× PBS and 10% ethanol was used for dilution and as the negative control. For the Aβ/QDAβ mixture, frozen Aβ42 stock solution in DMSO was thawed at room temperature and mixed by pipetting. QDAβ, 10× PBS, and ultrapure water were then added to prepare an Aβ solution containing 50 µM Aβ42, 50 nM QDAβ, and 1× PBS. The solution was centrifuged at 20,400× *g* for 1 min at 4 °C, and the supernatant was used for the assay. The Aβ solution was mixed with each food sample solution at a 1:1 ratio, yielding final concentrations of 25 µM Aβ42 and 25 nM QDAβ. For automated MSHTS, food samples and assay buffer were dispensed into 384-well plates, and five-fold serial dilutions were prepared using an automated workstation (JANUS G3, PerkinElmer, Waltham, MA, USA). Diluted samples were transferred to 1536-well black plates (782096, Greiner Bio-One, Kremsmünster, Austria) at 2.5 µL per well, with four replicate wells for each concentration. The Aβ/QDAβ solution was then dispensed at 2.5 µL per well and mixed with the sample solution. The plates were sealed to prevent evaporation and centrifuged at 1530× *g* for 5 min at 37 °C to remove bubbles and equalize the liquid surface. Fluorescence images were acquired before incubation using an inverted fluorescence microscope (ECLIPSE Ti-E, Nikon Corporation, Tokyo, Japan) equipped with a color CMOS camera (DS-Ri2, Nikon Corporation, Tokyo, Japan), a 4× objective lens, and a TRITC filter set. Images were obtained with the exposure conditions adjusted to complete imaging of all wells within 1 h. The plates were then incubated at 37 °C for 24 h, and fluorescence images were acquired again under the same conditions. For manual MSHTS used in follow-up or reference experiments, food extract samples were serially diluted five-fold, and soy sauce samples were serially diluted ten-fold in assay buffer. Each diluted sample was mixed with the Aβ/QDAβ solution at a 1:1 ratio, centrifuged at 10,000× *g* for 2 min at 4 °C to remove insoluble particles, and 5 µL of the supernatant was transferred to a 1536-well black plate. Fluorescence images were obtained before and after 24 h incubation at 37 °C using the same inverted fluorescence microscope described above.

#### 2.5.2. Quantification of Aβ42 Aggregation from MSHTS Images

Aβ42 aggregation was quantified from QDAβ fluorescence images using the standard deviation (SD) of fluorescence intensity as an index of aggregate formation. For automated MSHTS images, SD values were calculated from the central region of each well using NIS-Elements AR (version 4.51.00) software. For manual MSHTS images, the central 200 × 200 pixel region was analyzed using ImageJ (version 1.54g). The mean SD value was calculated from replicate wells for each sample concentration. Aggregation levels were normalized by defining the pre-incubation SD value as 0% and the post-incubation SD value of the Aβ42-alone control as 100%. Concentration–response curves were generated using a five-parameter logistic model in GraphPad Prism (version 11.0.1). EC_50_ was defined as the sample concentration at which the normalized aggregation level reached 50%. Lower EC_50_ values indicate higher Aβ42 aggregation-inhibitory activity.

### 2.6. Transmission Electron Microscopy

Aβ42 aggregates formed in the presence or absence of selected food samples were observed by TEM. Food sample solutions and the Aβ42/QDAβ solution were prepared as described for MSHTS. The Aβ42/QDAβ solution contained 50 µM Aβ42, 50 nM QDAβ, and 1× PBS. Each food sample solution was mixed with an equal volume of the Aβ42/QDAβ solution, and the Aβ42-alone control was prepared by mixing assay buffer with the Aβ42/QDAβ solution at the same ratio. Unless otherwise indicated, food samples were used at a final concentration of 0.2 mg/mL. The mixtures were centrifuged at 10,000× *g* for 2 min at 4 °C, aliquoted into PCR tubes, and incubated at 37 °C for 24 h. For negative staining, 10 µL of each sample solution was applied to a collodion-coated copper grid (U1005, EM Japan Co., Ltd., Tokyo, Japan) and allowed to adsorb for 5 min at room temperature. The excess solution was removed with filter paper. The grid surface was then briefly contacted with drops of ultrapure water twice, followed by 1% phosphotungstic acid (79690-25G, Sigma-Aldrich, St. Louis, MO, USA) twice. After removal of excess staining solution with filter paper, the grids were air-dried for 5 min. TEM observations were performed using an H-7600 transmission electron microscope (Hitachi High-Technologies, Tokyo, Japan) operated at 60 kV. TEM observation was used as a qualitative morphological assessment to support the aggregation assay results; quantitative fibril length or aggregate density analysis was not performed.

### 2.7. ThT Assay

Aβ42 fibrillar aggregation was evaluated using a ThT fluorescence assay. Food extract samples were diluted from 2 mg/mL stock solutions with assay buffer (10% ethanol and 1× PBS) by five-fold serial dilution to obtain five concentrations. Soy sauce samples were diluted separately by ten-fold serial dilution to obtain five concentrations. Assay buffer was used as the Aβ42-alone control. Aβ42 solution was prepared immediately before use. Frozen 1 mM Aβ42 stock solution in DMSO was thawed at room temperature and mixed by pipetting for 10 min. A premixed solution containing 10× PBS and ultrapure water was then added to the Aβ42 stock solution and mixed for 1 min to prepare an Aβ42 solution containing 50 µM Aβ42 and 1× PBS. Each diluted food sample solution was mixed with an equal volume of the Aβ42 solution, yielding a final Aβ42 concentration of 25 µM. Aliquots of the mixtures were dispensed into PCR tubes in triplicate and incubated at 37 °C for 24 h. Immediately before fluorescence measurement, ThT solution was prepared at 5 µM in 50 mM glycine-NaOH buffer (pH 8.5) using 100 µM ThT stock solution. ThT was purchased from FUJIFILM Wako Pure Chemical Corporation (202-01002, Osaka, Japan). After incubation, 190 µL of ThT solution was added to 10 µL of each sample mixture and mixed. The resulting 200 µL mixture was transferred to a 96-well plate (195-96F, Watson Co., Ltd., Tokyo, Japan), and fluorescence intensity was measured using a microplate reader (SH-9000Lab, Corona Electric Co., Ltd., Hitachinaka, Japan) with excitation at 455 nm and emission at 490 nm. EC_50_ values were estimated by fitting concentration–response curves using a five-parameter logistic model in GraphPad Prism. Parallel Aβ42-free control samples containing each food sample and ThT were measured under the same conditions. The raw fluorescence value of each Aβ42-free food-sample control was subtracted from the corresponding raw fluorescence value of the Aβ42-containing sample condition before generating concentration–response curves and estimating EC_50_ values.

### 2.8. MTT Assay

PC12 cells were cultured in high-glucose D-MEM containing L-glutamine, phenol red, and sodium pyruvate (041-30081, FUJIFILM Wako Pure Chemical Corporation, Osaka, Japan), supplemented with 10% fetal bovine serum (FBS; 536-90165, HyClone) and 0.5% penicillin–streptomycin (P4333, Sigma-Aldrich, St. Louis, MO, USA). Cells were maintained at 37 °C in a humidified atmosphere containing 5% CO_2_. For the MTT assay, 96-well tissue culture plates (3860-096, IWAKI, Shizuoka, Japan) were coated with poly-D-lysine (PDL). Poly-D-lysine hydrobromide (molecular weight 70,000–150,000; P0899-5mg, Sigma-Aldrich, St. Louis, MO, USA) was dissolved in ultrapure water at 5 mg/mL, filtered, aliquoted, and stored at 4 °C. Before cell seeding, the PDL stock solution was diluted to 0.1 mg/mL with sterile water, added to each well at 50 µL/well, and incubated for 5 min. After removal of the solution, each well was washed twice with 50 µL sterile water for 5 min each and air-dried at room temperature for 1 day. After drying, PC12 cells were seeded at 7000 cells/well in 200 µL culture medium and incubated for 4 h at 37 °C under 5% CO_2_. Nerve growth factor (NGF; mouse NGF 2.5S, 93928-24-6, Cosmo Bio Co., Ltd., Tokyo, Japan) was prepared in sterile PBS and added to each well to obtain a final concentration of 5 ng/mL. Cells were then treated with NGF for 24 h at 37 °C under 5% CO_2_ before Aβ42 exposure. This 24 h NGF treatment was used to prepare NGF-treated PC12 cells for exploratory cell-viability testing, rather than to establish a fully mature neuronal phenotype. After differentiation, the medium was removed, and 60 µL/well of sample/Aβ42 solution was carefully added to avoid detaching the cells. The final treatment solution contained 0.4, 0.08, or 0.016 mg/mL food sample and 2.5 µM Aβ42. Cells were incubated for 24 h at 37 °C under 5% CO_2_. After treatment, MTT solution prepared at 15 mg/mL in 1× PBS was added at 6 µL/well to 60 µL/well of cell-treatment solution, corresponding to a final working concentration of approximately 1.36 mg/mL, and cells were incubated for 4 h at 37 °C under 5% CO_2_. The supernatant was then removed as completely as possible without aspirating the formazan crystals. SDS solution (10% SDS in 0.01 M HCl) was added at 50 µL/well, and the plates were incubated overnight at 37 °C under 5% CO_2_ to solubilize the formazan. Absorbance at 570 nm was measured using a microplate reader (SH-9000Lab, Corona Electric Co., Ltd., Hitachinaka, Japan). Each condition was measured in triplicate wells (*n* = 3 wells per condition). For statistical comparison of the MTT assay, each Aβ42-containing food-sample condition was compared with the Aβ42-alone group using two-sided Welch’s *t*-test. To reduce sample-dependent interference with the MTT readout, Aβ42-free sample-matched control wells were measured in parallel for each food sample concentration. The absorbance value of each Aβ42-free sample-matched control was subtracted from the corresponding Aβ42-containing condition before normalization. Cell viability was calculated by normalizing the corrected absorbance to the DMSO control without Aβ42. The MTT assay was interpreted as an exploratory comparison of Aβ42-containing conditions with the Aβ42-alone group, rather than as an independent evaluation of the cytotoxicity or metabolic effects of each food sample alone.

### 2.9. Summary of Solvent Conditions

To improve reproducibility, the solvent conditions used in each assay are summarized here. In the MSHTS assay, food extract samples were prepared in 1× PBS containing 10% ethanol and 2% DMSO before mixing with the Aβ42/QDAβ solution at a 1:1 ratio. Thus, the aggregation reaction contained 25 µM Aβ42, 25 nM QDAβ, 5% ethanol, and 1× PBS. The Aβ42 stock solution contributed 2.5% DMSO to the final aggregation reaction, and food-extract stocks contributed additional sample-dependent DMSO up to 1% at the highest food-extract concentration. Soy sauce samples were prepared separately in 1× PBS containing 10% ethanol and mixed with the Aβ42/QDAβ solution at a 1:1 ratio. In the ThT assay, food extract and soy sauce samples were diluted in 1× PBS containing 10% ethanol and mixed with the Aβ42 solution at a 1:1 ratio, resulting in 25 µM Aβ42 and 5% ethanol during Aβ42 incubation. As in the MSHTS assay, the Aβ42 stock solution contributed 2.5% DMSO to the final Aβ42 incubation mixture, and food-extract stocks contributed additional sample-dependent DMSO up to 1% at the highest food-extract concentration. In the MTT assay, the final cell-treatment solution contained 2.5 µM Aβ42; 0.4, 0.08, or 0.016 mg/mL food sample; and 1.4% DMSO.

### 2.10. Statistical Analysis

Data are presented as mean ± SD unless otherwise indicated. In the automated MSHTS assay, each concentration was measured in four replicate wells. In the ThT assay, each concentration was measured in triplicate. In the MTT assay, each condition was measured in triplicate wells (*n* = 3 wells per condition). These measurements were technical replicates within the same experimental run, and independent biological replicate experiments were not performed. EC_50_ values estimated from MSHTS and ThT assays were compared descriptively using mean ± SD values and fold differences. Because these comparisons were intended to evaluate assay-level consistency rather than to test predefined statistical hypotheses, formal statistical tests and confidence interval estimation were not performed for EC_50_ comparisons. For the MTT assay, each Aβ42-containing food-sample condition at each concentration was compared with the Aβ42-alone group using two-sided Welch’s *t*-test. Welch’s *t*-test was used because equal variances between groups were not assumed. No correction for multiple comparisons was applied; therefore, the statistical results of the MTT assay were interpreted as exploratory. Exact *p*-values for the MTT assay comparisons are provided in Table S4.

## 3. Results

### 3.1. Primary Automated MSHTS Identifies Food Samples with Inhibitory Activity Against Aβ42 Aggregation

**Figure 1:**
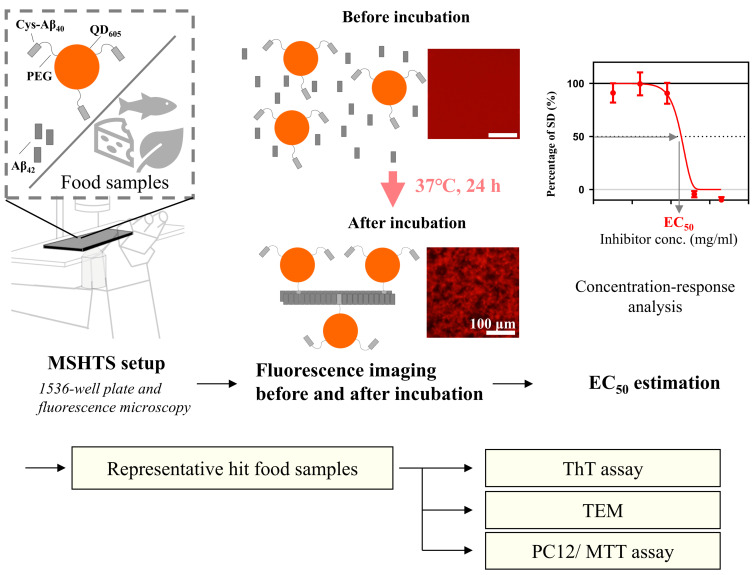
Overview of the study design and automated MSHTS workflow for identifying food-derived inhibitors of Aβ42 aggregation. Food samples, including extracts and selected food-derived samples, were evaluated using an automated microliter-scale high-throughput screening (MSHTS) assay. Aβ42 was co-incubated with QDAβ in 1536-well plates, and fluorescence images were acquired before and after incubation. Aggregation was quantified by the standard deviation (SD) of fluorescence intensity, and concentration–response curves were used to estimate EC_50_ values. Representative hit food samples were subsequently subjected to ThT assay, TEM analysis, and cell-based evaluation using differentiated PC12 cells.

Using automated MSHTS, we performed primary screening of food samples for inhibitory activity against Aβ42 aggregation. This image-based assay co-aggregates QDAβ with unlabeled Aβ42 and evaluates the amount of Aβ42 aggregates from the heterogeneity of fluorescence intensity in microscopic images (Figure 1). Before aggregation, QDAβ is dispersed relatively uniformly in solution, producing nearly homogeneous fluorescence images. After incubation at 37 °C for 24 h, incorporation of QDAβ into Aβ42 aggregates increases the heterogeneity of fluorescence intensity. Therefore, in this study, Aβ42 aggregation was quantified using the standard deviation (SD) of image intensity as an index, and the Aβ42 aggregation-inhibitory activity of each sample was evaluated by estimating EC_50_ values from concentration–response curves. By applying this assay to 1536-well plates and an automated dispensing system, we established an automated MSHTS workflow that enabled comparison of many food samples under uniform conditions. Rosmarinic acid was included as an assay-validation control and showed a concentration-dependent reduction in Aβ42 aggregation-related SD values in the MSHTS assay (Figure S4A).

The resulting screening data were used to rank samples by 1/EC_50_, examine the distribution of active samples across broad food categories, and select representative hit foods for subsequent validation by ThT assay, TEM observation, and MTT assay (Figure 1).

**Figure 2:**
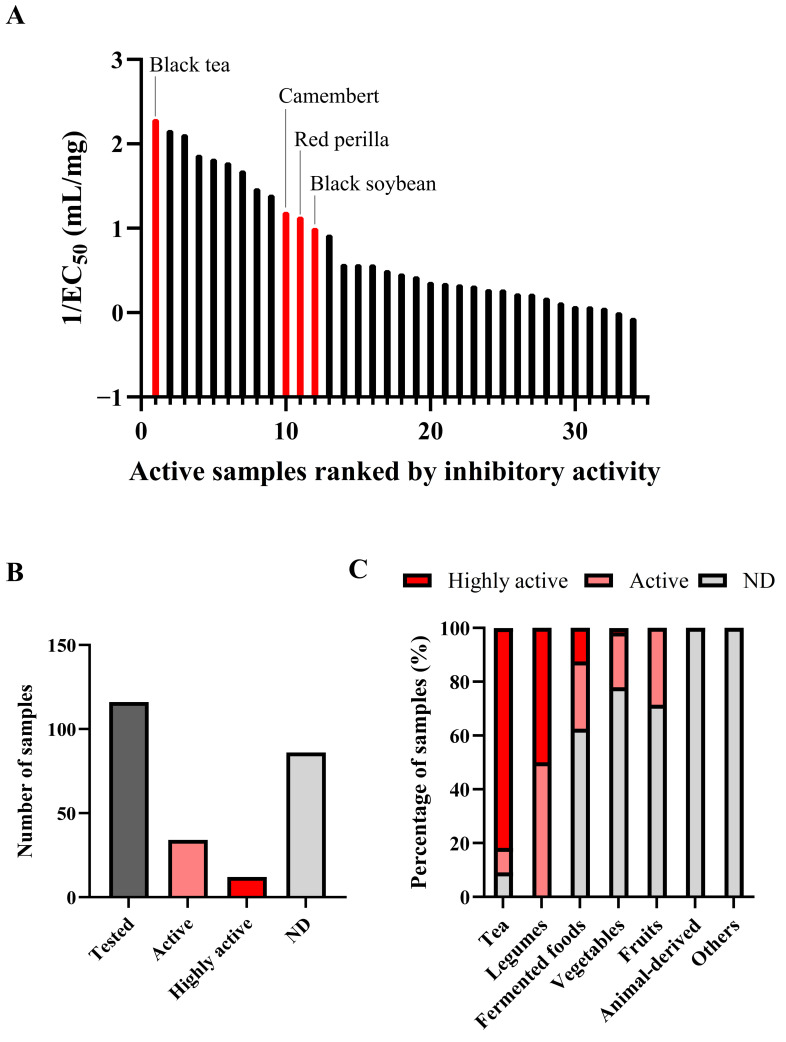
Primary automated MSHTS identifies food samples with inhibitory activity against Aβ42 aggregation. (**A**) Ranked distribution of inhibitory activity among active samples identified in the automated screening set. Inhibitory activity is shown as 1/EC_50_ (mL/mg) on a logarithmic scale, with higher values indicating stronger inhibitory activity. Representative hit food samples selected for downstream analyses are highlighted. (**B**) Summary of screening outcomes in the automated screening set, including the numbers of tested, active, highly active, and not detected (ND) samples. (**C**) Distribution of ND, active, and highly active samples across major food categories, shown as percentages within each category. Highly active samples were defined as those with 1/EC_50_ ≥ 10 mL/mg. The Tea category included one coffee sample, and the Others category mainly consisted of processed foods, including vegetable/fruit juices and canned foods. Because the number of samples differed among categories, this panel is intended as a descriptive summary of the screened sample set rather than a statistical comparison among food categories.

As a result of the primary screening by automated MSHTS, Aβ42 aggregation-inhibitory activity varied markedly among food samples, and detectable Aβ42 aggregation-inhibitory activity was observed in 34 of the 120 food-sample preparations (Figure 2A; Table S1). When the samples showing detectable Aβ42 aggregation-inhibitory activity were compared using 1/EC_50_ as an activity index, several samples, including Black tea, showed high inhibitory activity (Figure 2A). Samples with 1/EC_50_ ≥ 10 mL/mg were defined as highly active, and 12 samples met this criterion (Figure 2B).

Next, the food samples were classified into broad food categories based on their common food use, major edible part, or food group, and the distribution of Aβ42 aggregation-inhibitory activity within each category was summarized descriptively (Figure 2C, Table S1). Highly active samples were frequently observed among tea-related samples within the present screening set. Because the number of samples differed among categories, categories with small sample numbers, such as Legumes, were interpreted cautiously and were not used to infer category-wide trends. Highly active samples were observed in Tea (9/11), Vegetables (1/59), Fermented foods (1/8), and Legumes (1/2), whereas no highly active samples were observed in Fruits (0/21), Animal-derived foods (0/5), or Others (0/14).

Based on these results, Black tea, Camembert, Red perilla, and Black soybean were selected as representative hit foods for subsequent validation experiments. These samples were not selected solely on the basis of EC_50_ ranking. Rather, they were selected because they showed relatively high Aβ42 aggregation-inhibitory activity in the automated MSHTS assay and represented different food categories or food matrices, including tea-related samples, fermented dairy products, vegetables/herbs, and legumes. This selection allowed us to evaluate whether the screening hits could be supported by orthogonal assays across compositionally distinct food samples, rather than focusing only on the highest-ranked samples (Figure 2A).

**Figure 3:**
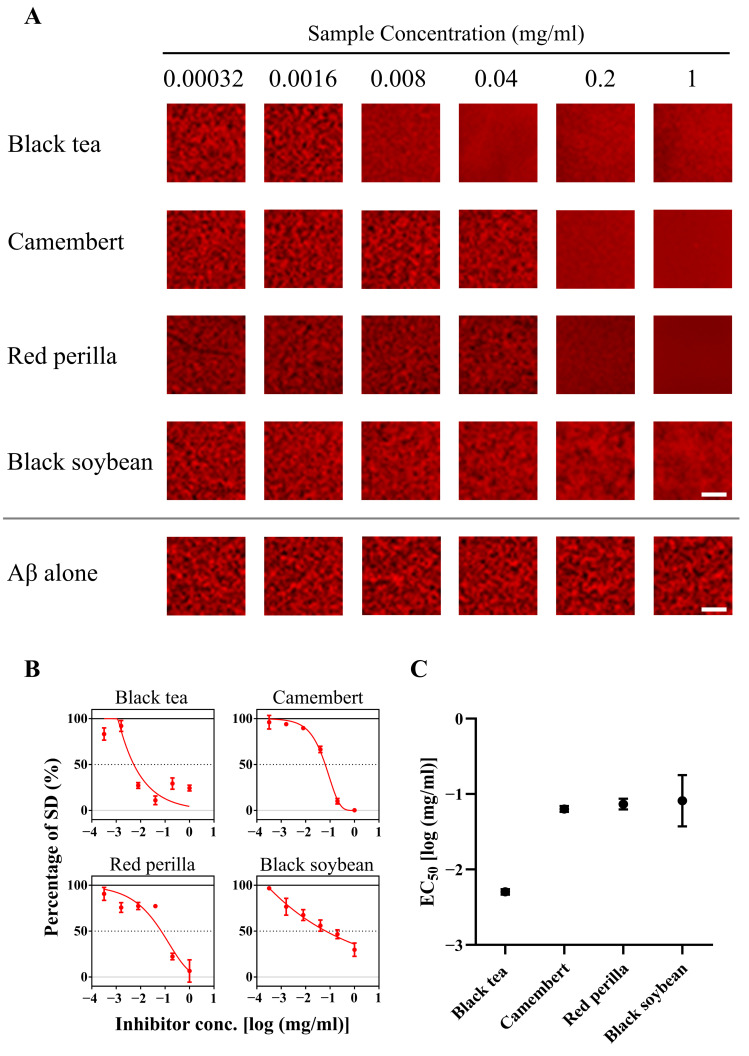
Representative MSHTS fluorescence images and concentration–response profiles of representative hit food samples. (**A**) Representative fluorescence images of Aβ42 aggregates in the absence or presence of representative hit food samples at the indicated concentrations. Concentrations are arranged from left to right in ascending order, corresponding to decreasing aggregate formation. Scale bar, 100 µm. (**B**) Concentration–response curves derived from the SD values of fluorescence images for each representative hit food sample, shown in separate panels. Data points represent mean ± SD from four wells at each concentration. (**C**) Estimated EC_50_ values of representative hit food samples determined by automated MSHTS. EC_50_ values are shown on a logarithmic scale and are presented as mean ± SD.

For these representative hit foods, fluorescence images obtained by automated MSHTS are shown as representative images (Figure 3A). In the Aβ42-alone condition, marked heterogeneity in fluorescence intensity was observed after incubation. In contrast, in the presence of representative hit foods, the fluorescence images became more homogeneous with increasing sample concentration, visually confirming suppression of Aβ42 aggregate formation. The concentration–response curves and estimated EC_50_ values derived from these images are shown in Figure 3B,C. Although the estimated EC_50_ value for Black soybean showed a relatively large error range, this likely reflected its gradual concentration–response profile, in which the transition around the 50% response level was less sharply defined than those of the other representative samples. Overall, these image-derived data were generally consistent with the primary screening results shown in Figure 2 and indicated concentration-dependent Aβ42 aggregation-inhibitory activity of the representative hit foods.

In addition, to support the interpretation of food categories that showed high activity in the primary screening, selected follow-up/reference samples were further evaluated by MSHTS (Figure S1, Table S1). In the Tea category, Green tea and Roasted green tea also showed strong Aβ42 aggregation-inhibitory activity, consistent with the observation that tea-related samples were frequently detected among highly active samples in the present screening set. Soy sauce and raw soy sauce, which were evaluated to confirm consistency with previous findings, also showed Aβ42 aggregation-inhibitory activity, in agreement with previously reported inhibitory activity of soy sauce-related samples against Aβ42 aggregation [27]. These follow-up/reference samples were not included in the primary automated MSHTS ranking and were treated as supplementary data for interpreting representative hit selection.

### 3.2. Orthogonal Validation of Selected Hit Samples by ThT Assay and TEM

**Figure 4:**
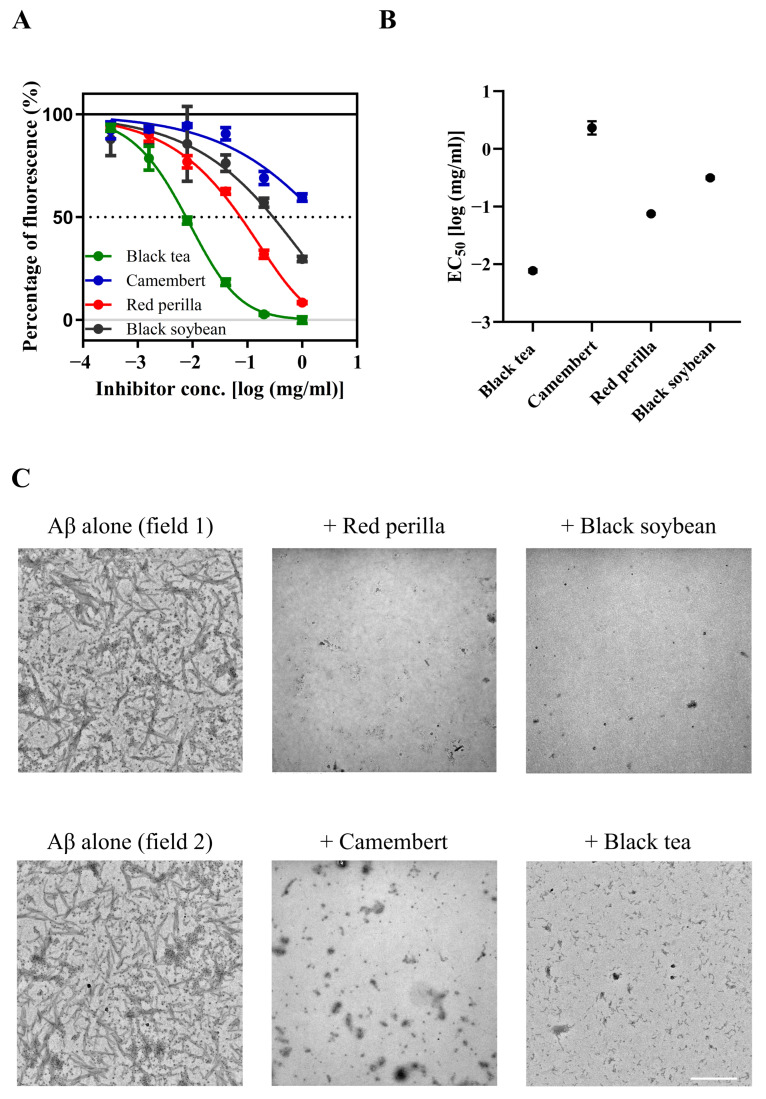
Orthogonal validation of representative hit food samples by ThT assay and TEM. (**A**) Representative inhibition curves obtained by ThT assay for representative hit food samples. Data points represent mean ± SD from three measurements at each concentration. (**B**) EC_50_ values estimated from ThT assay, shown on a logarithmic scale. Values are presented as mean ± SD. (**C**) Representative TEM images of Aβ42 incubated alone or with representative hit food samples. Two representative TEM fields of Aβ42 alone acquired at the same magnification are shown. In the absence of food samples, fibrillar Aβ42 aggregates were observed, whereas aggregate morphology was reduced or altered in the presence of representative hit food samples. All images were acquired at 8000× magnification. Scale bar, 500 nm.

The automated MSHTS method used for primary screening is a high-throughput adaptation of an Aβ aggregation visualization and quantification method based on QDAβ [24,26]. Although this method is suitable for primary screening, it is an image-based indirect assay and therefore requires validation using orthogonal approaches. We therefore used the ThT assay and TEM observation to examine whether the representative hit foods identified by screening inhibited Aβ42 aggregation (Figure 4A,B). Rosmarinic acid also reduced Aβ42 aggregation-related ThT fluorescence in a concentration-dependent manner (Figure S4B), confirming that the ThT assay detected a known Aβ aggregation inhibitor under the present conditions. All representative hit foods reduced ThT fluorescence, indicating a tendency to suppress the formation of ThT-positive Aβ42 fibrillar aggregates. The Aβ42-free food-sample controls generally showed ThT fluorescence signals of approximately 10% of the Aβ42-alone signal after 24 h incubation, and these background signals were subtracted before curve fitting. Comparison of EC_50_ values obtained by MSHTS and ThT assay showed that the values for Black tea, Red perilla, and Black soybean were generally consistent, whereas Camembert showed an exceptional difference of more than one order of magnitude (Table S2). This discrepancy suggests that Camembert may affect aggregate species detected by MSHTS and ThT assay differently.

Next, the morphology of Aβ42 aggregates was qualitatively evaluated by TEM observation (Figure 4C and Figure S2). Under the Aβ42-alone condition, fibrillar aggregates were observed in both fields acquired at the same magnification. In contrast, in the presence of the representative hit foods, TEM images showed fewer fibrillar aggregates than under the Aβ42-alone condition, along with fibril fragmentation or other morphological changes. These qualitative observations were consistent with the conclusion that the representative hit foods affected Aβ42 aggregation.

Taken together, the Aβ42 aggregation-inhibitory activity of the representative hit foods selected by automated MSHTS was generally supported by ThT assay and TEM observation. These results indicate that the hits obtained by MSHTS were not limited to image-based primary screening outcomes, but also affected Aβ42 aggregation in terms of ThT-positive aggregate formation and aggregate morphology.

### 3.3. Selected Hit, Follow-Up, and Reference Samples Show Increased Cell Viability in Aβ42-Treated Differentiated PC12 Cells

**Figure 5:**
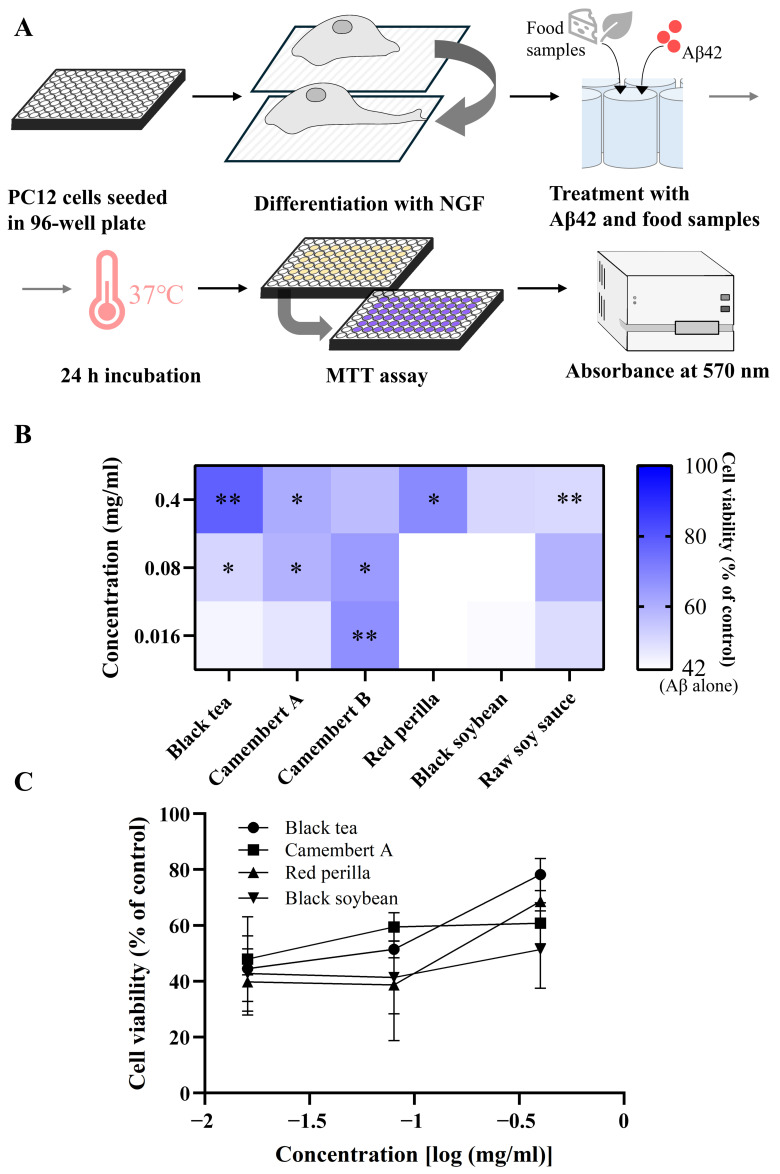
Effects of representative hit food samples on cell viability in Aβ42-treated differentiated PC12 cells. (**A**) Schematic overview of the cell-based assay. PC12 cells were seeded, differentiated with NGF, treated with Aβ42 and food samples, incubated for 24 h, and then subjected to MTT assay. Cell viability was estimated by measuring absorbance at 570 nm and expressed relative to the DMSO-only control. (**B**) Heat map showing the effects of selected food samples on cell viability in Aβ42-treated differentiated PC12 cells. Color intensity represents mean cell viability (% of the DMSO-only control). All sample conditions include Aβ42 unless otherwise indicated. Asterisks indicate nominal *p*-values for increases in cell viability compared with the Aβ42-alone group (* *p* < 0.05, ** *p* < 0.01; two-sided Welch’s *t*-test). (**C**) Representative dose–response plots showing the effects of selected food samples on cell viability in differentiated PC12 cells treated with Aβ42. Data are presented as mean ± SD from three replicate wells.

Next, we performed an MTT assay using differentiated PC12 cells to evaluate the effects of representative hit foods and selected follow-up/reference samples on Aβ42-induced reduction in cell viability (Figure 5). Rosmarinic acid was included as an assay-validation control for the MTT assay, and rosmarinic acid-alone controls were measured to evaluate its direct effect on the MTT readout (Figure S4C). Cell viability was calculated by defining the DMSO control as 100% and was compared with the Aβ42-alone group. The values shown were calculated after subtraction of sample-matched Aβ42-free blanks containing differentiated PC12 cells and the corresponding food sample concentration. Mean cell viability in the presence of each food sample is shown as a heatmap in Figure 5B. For Camembert, follow-up samples from the same food category, distinct from the Camembert sample identified by primary automated MSHTS, were used in this analysis. These Camembert follow-up samples were separately evaluated by MSHTS and showed lower Aβ42 aggregation-inhibitory activity than the Camembert hit obtained in the primary screening (Table S3). The MTT results for the Camembert follow-up samples were therefore interpreted as exploratory data for related fermented dairy samples, rather than as direct cell-based validation of the primary Camembert hit. Raw soy sauce was not included in the primary automated screening set and was treated as a reference sample in this analysis to examine consistency with previous findings.

The effects on Aβ42-induced reduction in cell viability differed among food samples. Black tea, Red perilla, and the Camembert follow-up samples showed increased cell viability compared with the Aβ42-alone group under some conditions. In contrast, although increased cell viability was observed for the Camembert follow-up samples and raw soy sauce under some conditions, these changes did not necessarily show a clear concentration-dependent pattern. Black soybean showed Aβ42 aggregation-inhibitory activity in both MSHTS and ThT assays but did not show a clear improvement in cell viability in differentiated PC12 cells (Figure 5B,C).

These results suggest that some food samples with Aβ42 aggregation-inhibitory activity may partially attenuate Aβ42-induced reduction in cell viability under specific conditions in differentiated PC12 cells. However, the effects were not consistently concentration-dependent across samples, and aggregation-inhibitory activity and improvement in cell viability did not always correspond, suggesting that cellular responses cannot be explained solely by the strength of aggregation-inhibitory activity obtained in the primary screening.

## 4. Discussion

In this study, we used automated MSHTS to systematically evaluate the Aβ42 aggregation-inhibitory activity of food samples across multiple food categories. The results showed that Aβ42 aggregation-inhibitory activity varied markedly among food samples and that some samples exhibited high inhibitory activity. In particular, Black tea, Camembert, Red perilla, and Black soybean were selected as representative hit foods for subsequent analyses. The significance of this study lies in the primary selection of Aβ42 aggregation-modulating candidates by comparing diverse food samples on the same automated MSHTS platform, rather than evaluating a single food or a specific food category. Compared with previous high-throughput QDAβ/MSHTS studies that established the image-based assay and automated screening workflow [24,25,26], and previous food-, plant-, or natural-product-focused applications that evaluated specific food groups or extract libraries [27,28,29], the contribution of the present study is the use of this established platform for standardized cross-category comparison of diverse food samples, followed by downstream validation using independent aggregation assays and a cell-based assay. Foods are complex multicomponent matrices, and their measured Aβ42 aggregation-inhibitory activity may vary depending on component composition, processing conditions, extraction conditions, and other factors. Therefore, cross-category screening such as that performed in this study provides a useful basis for efficiently selecting candidate samples that can be further investigated in functional food research and active component analysis. Previous reviews have described Aβ aggregation and amyloid fibrillation as targets of aggregation-modulating agents, including small molecules, naturally occurring compounds, and nanomaterials [31].

Several of the representative hits identified in this study were also consistent with previous findings on food-derived or naturally occurring Aβ aggregation modulators. In the automated MSHTS, tea samples, including Black tea, accounted for most of the highly active samples, and additional validation showed that Green tea and Roasted green tea exhibited comparable Aβ42 aggregation-inhibitory activity (Figure 2A and Figure S1, Table S1). Tea consumption has been suggested to be associated with AD prevention in several studies [32,33], and polyphenolic components in tea have also been reported to possess Aβ aggregation-inhibitory activity [34]. In addition, among Black tea samples with different storage conditions, samples incubated at 37 °C for 3 days showed higher Aβ42 aggregation-inhibitory activity, and N_2_-filled conditions also tended to slightly increase this activity (Table S1). Tea processing and fermentation are known to alter the phytochemical profile of tea, including the formation of black-tea polyphenols such as theaflavins [35]. In addition, theaflavins have been reported to inhibit Aβ fibrillogenesis [36]. These findings suggest that the Aβ42 aggregation-inhibitory activity of tea samples may be influenced not only by food type but also by component changes associated with storage or processing conditions.

For Camembert, fermented dairy-derived peptides and fermented foods have been reported to affect neural function [37,38], suggesting that components generated during fermentation may be relevant to AD-related biological activity. For Black soybean, soybean isoflavones have been reported to alleviate Aβ burden and neuroinflammation in AD models [39,40]. In addition, the inclusion of Red perilla among the representative hits is consistent with previous reports on the Aβ aggregation-inhibitory activity of *Perilla frutescens* var. *crispa* leaf extracts [28].

Although the present study did not identify the active components responsible for the observed effects, several possible mechanisms may explain the Aβ42 aggregation-inhibitory activity of the representative hit foods. Food-derived polyphenols may influence Aβ42 assembly in a structure-dependent manner, including modulation of β-sheet-rich fibril formation and interactions with aggregation-prone peptide species [34]. In addition, polyphenols have been discussed as multifunctional compounds with antioxidant, metal-chelating, and anti-amyloidogenic properties, which may indirectly affect Aβ aggregation and Aβ-associated cellular responses [41].

At the level of individual representative hits, these considerations suggest distinct candidate component classes for future active-component analysis. For Black tea, the catechin/theaflavin-rich tea polyphenol axis discussed above represents a plausible candidate class, consistent with the strong activity of Black tea in MSHTS and the supporting ThT/TEM results [34,35,36]. For Red perilla, rosmarinic acid and other polyphenolic constituents are plausible candidates related to the observed Aβ42 aggregation-inhibitory activity [42]. For Black soybean, isoflavones and anthocyanins are plausible candidates related to the observed Aβ42 aggregation-inhibitory activity, because soybean isoflavones have been reported to inhibit Aβ fibrillization and oligomerization in vitro, and anthocyanin-rich extracts or anthocyanin compounds have been reported to modulate Aβ aggregation and Aβ-associated toxicity [43,44]. For Camembert, dairy- or fermentation-derived peptides, such as tryptophan-related dipeptides and β-lactolin, may be considered candidate component classes, although their presence in the tested extract and direct involvement in Aβ42 aggregation inhibition require future verification [37,38]. These hit-specific considerations suggest that the inhibitory activity detected in this study may reflect multiple component-dependent mechanisms rather than a single common mechanism shared by all hit foods.

On the other hand, this study also showed that food groups attracting attention in functional food research do not necessarily exhibit Aβ42 aggregation-inhibitory activity. In the Vegetables category, Red perilla was the only highly active sample among 59 items, and no Aβ42 aggregation-inhibitory activity was detected in more than 70% of the samples (Figure 2C). In the Fruits category, no highly active sample was identified, and more than 70% of the samples were classified as ND. In addition, processed foods, such as vegetable and fruit juices, which accounted for a large proportion of the Others category, did not show detectable Aβ42 aggregation-inhibitory activity in this assay system. Based on these results, the previously reported inverse association between fruit and vegetable intake and cognitive impairment risk [45] may involve multiple pathways other than direct inhibition of Aβ42 aggregation, such as antioxidant activity, anti-inflammatory effects, metabolic regulation, vascular function, and gut microbiota-related mechanisms [46,47]. Furthermore, in the Animal-derived category, including fish and meat, no samples showed Aβ42 aggregation-inhibitory activity in automated MSHTS (Figure 2C). Fish and meat intake has been reported to be associated with cognitive function measures, whereas its association with incident dementia risk appears limited [48]. These findings suggest that even if these food groups influence cognitive function, their effects are not necessarily manifested as direct inhibition of Aβ42 aggregation. Taken together, the present screening results may provide a basis for distinguishing between the cognitive function-related effects of foods and their direct inhibitory effects on Aβ42 aggregation.

The Aβ42 aggregation-inhibitory activities of representative hit foods identified by the automated MSHTS method were generally supported by the ThT assay and TEM observation (Figure 4). In the ThT assay, the formation of ThT-positive aggregates was reduced, and TEM observation also showed a decrease in the fibrillar structures observed under the Aβ42-alone condition. In addition, the EC_50_ values estimated by the MSHTS method and the ThT assay were within a similar concentration range for many samples (Table S2). For example, for Black tea, Red perilla, Green tea, Roasted green tea, and raw soy sauce, the EC_50_ values estimated by the two methods differed by approximately two-fold or less, and even for Black soybean, the difference was limited to approximately three-fold. In contrast, the EC_50_ values obtained by the two methods did not completely coincide, with Camembert showing an approximately 36-fold difference. This discrepancy may be attributable to differences in the aggregate species detected by the two methods. The MSHTS method mainly reflects relatively large QDAβ-containing aggregates that are detected as spatial heterogeneity in fluorescence microscopy images [24], whereas the ThT assay may reflect a broader range of aggregate species, from soluble or small ThT-positive assemblies that are difficult to clearly detect in microscopy images to mature fibrils [49]. Taken together, the Aβ42 aggregation-inhibitory activities of the representative hit foods were generally confirmed by the ThT assay and TEM observation, consistent with the trends indicated by the MSHTS method. Therefore, rather than indicating the superiority of one assay over another, the MSHTS method, ThT assay, and TEM observation can be positioned as complementary approaches for evaluating different aspects of Aβ42 aggregation behavior.

In the cell-based evaluation, many of the food samples tested in the MTT assay showed increased cell viability in Aβ42-treated cells, at least within some concentration ranges (Figure 5 and Figure S3). This result indicates that some of the candidate Aβ42 aggregation modulators identified by the MSHTS method may also affect Aβ42-induced cellular responses. However, the strength of Aβ42 aggregation-inhibitory activity did not completely correspond to the improvement in cell viability. Black soybean showed Aβ42 aggregation-inhibitory activity in both the MSHTS method and the ThT assay, but did not show a clear improvement in cell viability in the MTT assay. In addition, Camembert A and B were follow-up samples different from the Camembert hit identified in the primary automated MSHTS screen, and showed lower Aβ42 aggregation-inhibitory activity than the primary hit (Table S3). Nevertheless, increased cell viability was observed under some conditions. These results suggest that Aβ42 aggregation-inhibitory activity may serve as a useful primary indicator for predicting cellular responses, but that it alone cannot fully explain the cellular effects of food samples. This discrepancy may be attributable not only to the total amount of Aβ42 aggregates, but also to the properties of aggregate species involved in cytotoxicity and to the cellular effects of the food samples themselves. Because Aβ cytotoxicity can depend on the properties of aggregate species such as soluble oligomers, Aβ42 aggregate species that contribute to cytotoxicity may remain even when aggregation-inhibitory activity is detected by the MSHTS method or the ThT assay [50]. On the other hand, although food-derived polyphenols generally exhibit antioxidant activity, they have also been reported to exert pro-oxidant cellular effects under cell culture conditions, for example through H_2_O_2_ generation [51,52]. Therefore, changes in cell viability induced by food samples may reflect not only Aβ42 aggregation-inhibitory activity, but also both qualitative changes in Aβ42 aggregate species and the cellular effects of the food components themselves. Thus, Aβ42 aggregation-inhibitory activity should be regarded as a primary screening indicator for selecting food-derived candidates for further evaluation, rather than as a direct predictor of cellular efficacy. The present results indicate that evaluating both Aβ42 aggregation-inhibitory activity and the cellular effects of food components themselves is important for narrowing down food-derived candidates that modulate Aβ42 aggregation.

This study has several limitations. First, the food sample set used in this study was not designed to comprehensively or randomly represent each food category. Therefore, although the category-wise distribution of Aβ42 aggregation-inhibitory activity may suggest food groups that are more frequently represented among active samples, it does not allow conclusions regarding the efficacy or inefficacy of entire food categories. Accordingly, the category-wise analysis in this study should be interpreted not as evidence of general superiority or inferiority among food categories, but as the distribution of candidate foods within the sample set evaluated under the same conditions. In addition, because this study evaluated food extracts, it remains unclear which components, or groups of components, were responsible for the observed inhibitory activity. Moreover, preliminary compositional analyses, such as total polyphenol content, catechin or theaflavin levels, isoflavone content, or peptide fraction analysis, were not performed in this study. Therefore, future studies should combine the MSHTS-based screening platform with compositional profiling and fractionation analyses to identify the active components or component combinations responsible for the observed Aβ42 aggregation-modulating activity. In particular, food extracts may contain diverse constituents, including polyphenols, proteins, peptides, carbohydrates, organic acids, and salts; therefore, the observed effects cannot be interpreted as the action of a single component. Furthermore, the effective concentrations observed in the present assays should be interpreted as concentrations of crude food extracts or food-derived samples used for in vitro primary screening and follow-up validation, rather than as concentrations expected to be achieved through dietary intake. Because the tested samples were not purified compounds, the concentrations of the active constituents responsible for the observed effects are unknown. In addition, this study was based on purified Aβ42 aggregation assays and a cell-based assay under defined in vitro conditions, and did not evaluate digestion, intestinal absorption, metabolism, systemic bioavailability, blood–brain barrier penetration, or concentrations achievable in vivo. The reported variability reflects technical replicate measurements within a single experimental run and does not capture inter-experimental reproducibility. This limitation is particularly important for the MTT assay. Thus, the mechanistic bridge from in vitro aggregation inhibition to dietary preventive potential requires future identification of active constituents and evaluation of their digestion, absorption, metabolism, tissue distribution, and in vivo activity. Therefore, the present results should be positioned as candidate-level findings indicating that food-derived samples can affect Aβ42 aggregation under in vitro assay conditions, rather than as direct evidence for dietary efficacy or preventive effects in vivo.

## 5. Conclusions

This study supports a screening-to-validation workflow for prioritizing food-derived candidates that modulate Aβ42 aggregation. Screening of 120 food-sample preparations derived from 115 food items by automated MSHTS revealed substantial variation in Aβ42 aggregation-inhibitory activity, with highly active samples frequently detected among tea-related samples within the present screening set. Representative hits, including Black tea, Camembert, Red perilla, and Black soybean, showed concentration-dependent inhibition in MSHTS, and these effects were supported by ThT assay and TEM observation. MTT assay further showed that some selected samples increased cell viability in Aβ42-treated cells at some concentrations, while also indicating that MTT-based cellular responses cannot be predicted solely from aggregation-inhibitory activity. However, because the reported variability reflects technical replicate measurements within a single experimental run and does not capture inter-experimental reproducibility, these MTT results should be interpreted as exploratory findings requiring future confirmation. Thus, the combined assays support the use of automated MSHTS for primary candidate prioritization, followed by orthogonal aggregation assays and exploratory cell-based evaluation. This study provides a basis for future active component identification and mechanistic evaluation of food-derived Aβ42 aggregation modulators.

## Data Availability

The data presented in this study are available on request from the corresponding author.

## Supplementary Materials

The following supporting information can be downloaded at: https://www.mdpi.com/article/10.3390/foods15122108/s1, Table S1: Complete EC_50_ values for food samples evaluated by MSHTS; Figure S1: Additional MSHTS evaluation of selected follow-up and reference food samples; Table S2: Comparison of estimated EC_50_ values obtained from MSHTS and ThT assays; Figure S2: Additional TEM images of Aβ42 aggregates formed in the absence or presence of representative hit food samples; Table S3: MSHTS activity of follow-up Camembert samples used in the cell viability assay; Figure S3: Full MTT assay results for selected hit and follow-up food samples; Figure S4: Validation of MSHTS, ThT, and MTT assays using rosmarinic acid; Table S4: Statistical analysis of MTT assay results; Figure S5: Preliminary comparison of extraction solvents using tea-related samples.

## Abbreviations

The following abbreviations are used in this manuscript:

Aβ	Amyloid β
MSHTS system	microliter-scale high-throughput screening system
PBS	phosphate-buffered saline
QD	quantum dot
TEM	transmission electron microscope
ThT	thioflavin T
