# Supplementary material for: Cross-Category Screening of Food Samples for Amyloid-β42 Aggregation-Inhibitory Activity Using a Microliter-Scale High-Throughput Screening System with Quantum-Dot-Labeled Aβ
Source: Foods. 2026 Jun 11;15(12):2108. doi: 10.3390/foods15122108 (PMC13298185; doi:10.3390/foods15122108)
Supplement: Supplementary file 1 [file foods-15-02108-s001.zip › foods-4336128-supplementary.pdf]

Supplementary Materials

Cross-Category Screening of Food Samples for Amyloid-β42 Aggregation-Inhibitory Activity Using a Microliter-Scale High-Throughput Screening System with Quantum-Dot-Labeled Aβ

Table S1. Complete EC_{50} values for food samples evaluated by MSHTS, excluding follow-up Camembert samples. ND indicates samples for which EC_{50} could not be estimated under the tested conditions. Bold values in the $1/EC_{50}$ column indicate highly active samples, defined as $1/EC_{50} \geq 10$ mL/mg. †Follow-up or reference samples evaluated by manual MSHTS; all other samples were evaluated in the primary automated MSHTS. Follow-up Camembert samples are summarized separately in Table S3.

Sample ID	Sample name	Broad category	EC_{50} mean (mg/mL)	EC_{50} SD (mg/mL)	$1/EC_{50}$ mean (mL/mg)
S001	Bamboo shoot (boiled)	Vegetable	ND		
S002	Lotus root (boiled)	Vegetable	ND		
S003	Ginger	Vegetable	0.267	0.059	3.75
S004	Potato	Vegetable	ND		
S005	Sweet potato	Vegetable	ND		
S006	Carrot	Vegetable	ND		
S007	Turnip	Vegetable	ND		
S008	Burdock root	Vegetable	ND		
S009	Horseradish	Vegetable	ND		
S010	Daikon radish	Vegetable	ND		

Sample ID	Sample name	Broad category	EC_{50} mean (mg/mL)	EC_{50} SD (mg/mL)	$1/EC_{50}$ mean (mL/mg)
S011	Lily bulb	Vegetable	ND		
S012	Beet concentrate	Vegetable	ND		
S013	Shishito pepper	Vegetable	0.476	0.076	2.10
S014	Green pepper	Vegetable	ND		
S015	Okra	Vegetable	ND		
S016	Cucumber	Vegetable	ND		
S017	Eggplant	Vegetable	ND		
S018	Green bean	Vegetable	ND		
S019	Yellow bell pepper	Vegetable	ND		
S020	Red bell pepper	Vegetable	ND		
S021	Bitter melon	Vegetable	0.664	0.25	1.51
S022	Pumpkin	Vegetable	ND		
S023	Chili pepper	Vegetable	ND		
S024	Melon	Vegetable	ND		
S025	Sweet corn (boiled)	Vegetable	ND		
S026	Baby corn	Vegetable	0.806	0.20	1.32
S027	Snap pea	Vegetable	ND		
S028	Edamame	Legumes	0.265	0.12	3.77
S029	Black soybean	Legumes	0.0988	0.059	**10.1**
S030	Bean sprouts	Vegetable	ND		
S031	Daikon	Vegetable	ND		

Sample ID	Sample name	Broad category	EC_{50} mean (mg/mL)	EC_{50} SD (mg/mL)	$1/EC_{50}$ mean (mL/mg)
	radish sprouts				
S032	Broccoli sprouts	Vegetable	ND		
S033	Butterbur (boiled)	Vegetable	ND		
S034	Asparagus	Vegetable	0.840	0.049	1.19
S035	Garlic	Vegetable	ND		
S036	Onion	Vegetable	ND		
S037	Welsh onion	Vegetable	ND		
S038	Alpine leek	Vegetable	ND		
S039	Garlic chives	Vegetable	ND		
S040	Lettuce 1	Vegetable	0.119	0.053	8.41
S041	Lettuce 2	Vegetable	ND		
S042	Chinese cabbage	Vegetable	ND		
S043	Cabbage	Vegetable	ND		
S044	Red leaf lettuce	Vegetable	ND		
S045	Garland chrysanthe mum	Vegetable	0.531	0.21	1.88
S046	Komatsun a	Vegetable	1.15	0.72	0.868
S047	Bok choy	Vegetable	ND		
S048	Spinach	Vegetable	ND		
S049	Mizuna	Vegetable	ND		
S050	Celery	Vegetable	ND		
S051	Japanese parsley	Vegetable	ND		
S052	Red perilla	Vegetable	0.0743	0.012	**13.7**

Sample ID	Sample name	Broad category	EC_{50} mean (mg/mL)	EC_{50} SD (mg/mL)	$1/EC_{50}$ mean (mL/mg)
S053	Horseradish leaves	Vegetable	0.464	0.11	2.25
S054	Myoga ginger	Vegetable	0.463	0.013	2.16
S055	Broccoli	Vegetable	ND		
S056	Buna-shimeji mushroom	Vegetable	0.834	0.059	1.20
S057	King oyster mushroom	Vegetable	ND		
S058	Nameko mushroom	Vegetable	ND		
S059	Fresh wood ear mushroom	Vegetable	ND		
S060	Fresh shiitake mushroom	Vegetable	ND		
S061	Maitake mushroom	Vegetable	0.271	0.027	3.72
S062	Lemon	Fruits	0.312	0.028	3.21
S063	Sudachi	Fruits	0.990	0.31	1.01
S064	Orange	Fruits	ND		
S065	Yuzu	Fruits	ND		
S066	Red grapefruit	Fruits	ND		
S067	Satsuma mandarin	Fruits	ND		
S068	Japanese pear	Fruits	ND		
S069	Apple	Fruits	ND		
S070	Apricot	Fruits	0.432	0.027	2.31
S071	Plum	Fruits	ND		
S072	Japanese	Fruits	0.343	0.014	2.92

Sample ID	Sample name	Broad category	EC_{50} mean (mg/mL)	EC_{50} SD (mg/mL)	$1/EC_{50}$ mean (mL/mg)
	apricot				
S073	Cherry	Fruits	ND		
S074	Peach	Fruits	ND		
S075	Pineapple	Fruits	ND		
S076	Avocado	Fruits	0.598	0.22	1.67
S077	Banana	Fruits	ND		
S078	Persimmon	Fruits	ND		
S079	Red-fleshed kiwifruit	Fruits	ND		
S080	Green-fleshed kiwifruit	Fruits	ND		
S081	Red grape	Fruits	ND		
S082	Blueberry	Fruits	0.606	0.10	1.69
S083	Sockeye salmon	Animal-derived	ND		
S084	Tuna bone marrow	Animal-derived	ND		
S085	Pork belly	Animal-derived	ND		
S086	Chicken breast	Animal-derived	ND		
S087	Chicken thigh	Animal-derived	ND		
S088	Camembert	Fermented	0.0636	0.0056	**15.7**
S089	Red miso	Fermented	ND		
S090	Pickled daikon (takuan)	Fermented	ND		
S091	Pickled takana mustard	Fermented	0.588	0.20	1.90

Sample ID	Sample name	Broad category	EC_{50} mean (mg/mL)	EC_{50} SD (mg/mL)	$1/EC_{50}$ mean (mL/mg)
	greens				
S092	Pickled rakkyo	Fermented	ND		
S093	Nanban pepper	Fermented	1.33	1.1	1.15
S094	Shibazuke (eggplant)	Fermented	ND		
S095	Kimchi	Fermented	ND		
F001	Soy sauce†	Fermented	0.0729	0.024	**13.7**
F002	Raw soy sauce†	Fermented	0.0149	0.0036	**67.1**
S096	Sencha	Tea	0.0134	0.0019	**74.6**
S097	Gyokuro	Tea	ND		
S098	Bancha	Tea	0.00682	0.00085	**147**
S099	Oolong tea	Tea	0.00770	0.0020	**130**
S100	Black tea	Tea	0.00505	0.00044	**198**
S101	Coffee	Tea	0.0400	0.022	**25.0**
S102	Barley tea	Tea	0.371	0.064	2.70
S103	Black tea + N_2	Tea	0.0266	0.014	**48.4**
S104	Black tea - N_2	Tea	0.0350	0.0082	**29.8**
S105	Black tea + N_2 37 °C, 3 d	Tea	0.0163	0.0060	**66.7**
S106	Black tea - N_2 37 °C, 3 d	Tea	0.0181	0.0058	**60.5**
F003	Green tea†	Tea	0.0115	0.00062	**87.0**
F004	Roasted green tea†	Tea	0.00613	0.00043	**163**
S107	Konjac	Others	ND		

Sample ID	Sample name	Broad category	EC_{50} mean (mg/mL)	EC_{50} SD (mg/mL)	$1/EC_{50}$ mean (mL/mg)
S108	Tofu	Others	ND		
S109	Canned mackerel in water	Others	ND		
S110	Honey umeboshi	Others	ND		
S111	Vegetable juice	Others	ND		
S112	Carrot juice	Others	ND		
S113	Apple juice	Others	ND		
S114	Vegetable juice	Others	ND		
S115	Green juice	Others	ND		
S116	Grape juice	Others	ND		
S117	Pear juice	Others	ND		
S118	Peach juice	Others	ND		
S119	Fruit carrot	Others	ND		
S120	Tomato juice	Others	ND		

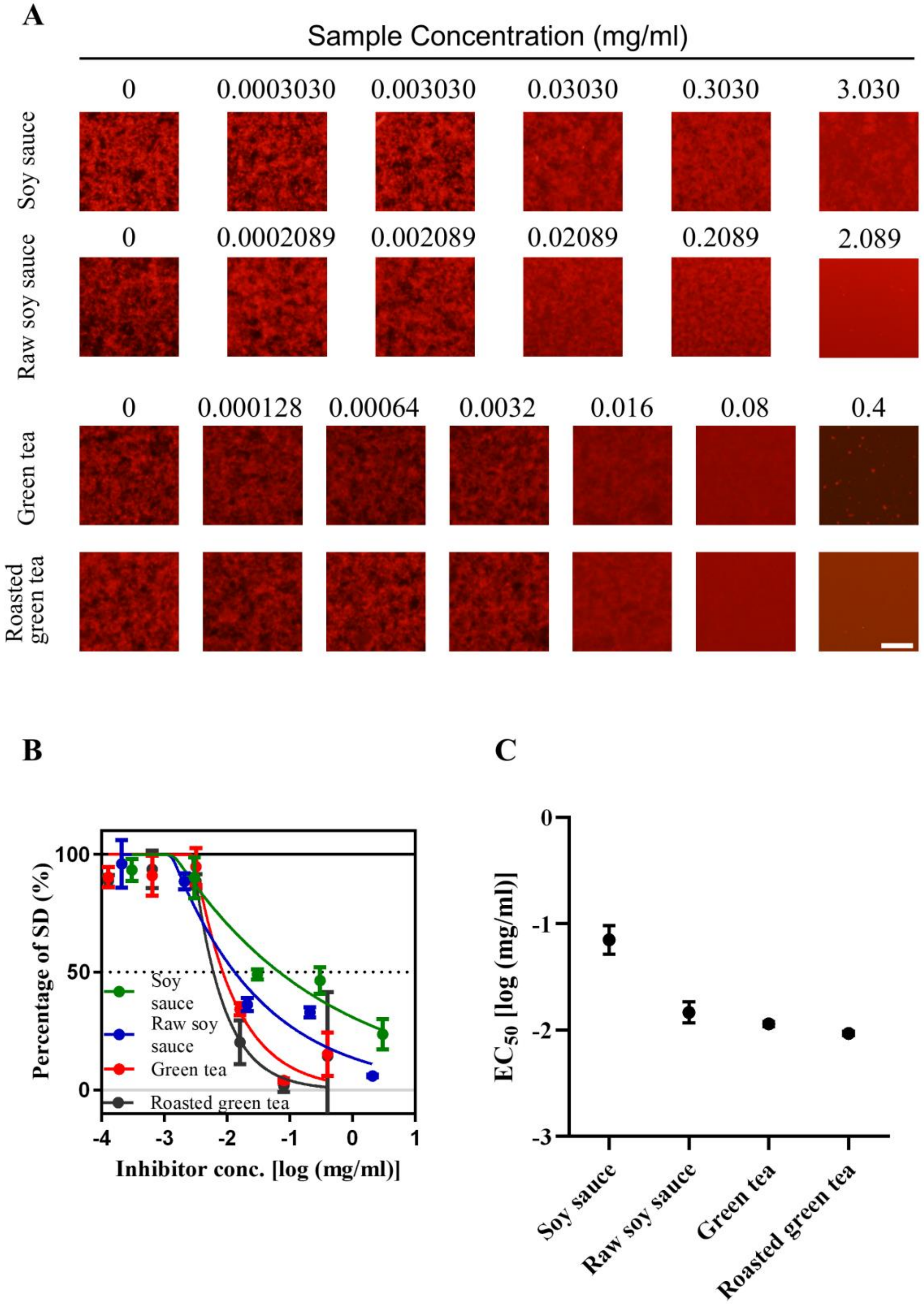
A
Sample Concentration (mg/ml)
0
0.0003030
0.003030
0.03030
0.3030
3.030
Soy sauce
0
0.0002089
0.002089
0.02089
0.2089
2.089
Raw soy sauce
0
0.000128
0.00064
0.0032
0.016
0.08
0.4
Green tea
Roasted green tea
B
Percentage of SD (%)
Soy sauce
Raw soy sauce
Green tea
Roasted green tea
Inhibitor conc. [log (mg/ml)]
C
EC50 [log (mg/ml)]
Soy sauce
Raw soy sauce
Green tea
Roasted green tea

Figure S1. Additional MSHTS evaluation of selected follow-up and reference food samples.

(A) Representative fluorescence images obtained by MSHTS for selected tea-related samples and soy sauce-related samples. Aβ42 aggregation was monitored using QDAβ fluorescence after incubation at 37 °C for 24 h. (B) Estimated EC_{50} values for the additional samples evaluated by MSHTS. Values are shown as mean ± SD. Green tea and Roasted green tea were evaluated as follow-up samples related to the Tea category. Soy sauce and raw soy sauce were evaluated as reference samples to examine consistency with previous findings. Scale bars, 100 μm.

Table S2. Comparison of estimated EC_{50} values obtained from MSHTS and ThT assays. Samples shown in the main figures are indicated in bold.

Sample	MSHTS EC_{50}	ThT EC_{50}	ThT/MSHTS Ratio
**Black tea**	5.05 ± 0.44 μg/mL	7.60 ± 0.60 μg/mL	1.5
**Camembert**	63.62 ± 5.63 μg/mL	2319.67 ± 0.71 μg/mL	36.5
**Red perilla**	74.32 ± 11.77 μg/mL	75.08 ± 2.35 μg/mL	1.0
**Black soybean**	98.79 ± 59.43 μg/mL	293.07 ± 52.46 μg/mL	3.0
Green tea	11.45 ± 0.62 μg/mL	10.99 ± 1.07 μg/mL	1.0
Roasted green tea	6.13 ± 0.43 μg/mL	9.12 ± 1.01 μg/mL	1.5
Raw soy sauce	14.91 ± 3.62 μg/mL	7.59 ± 0.27 μg/mL	0.5

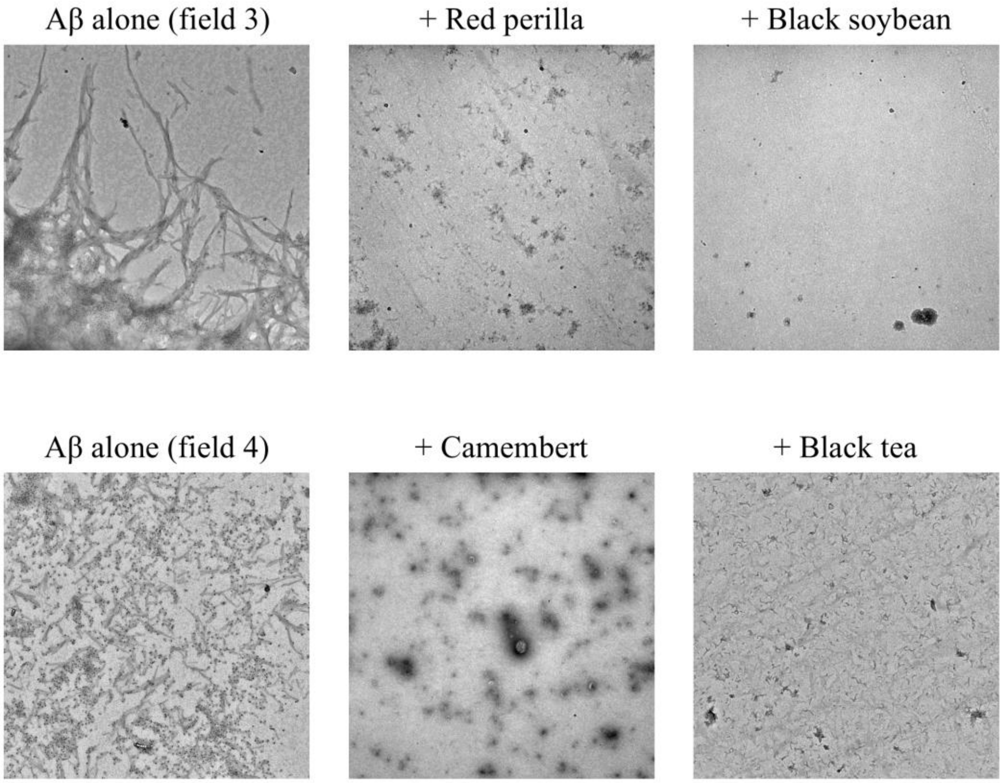

Figure S2. Additional TEM images of Aβ42 aggregates formed in the absence or presence of representative hit food samples.

Additional TEM fields are shown to complement the representative images in Figure 4C. Fields 3 and 4 show additional Aβ42-alone images acquired under the same condition as the Aβ42-alone images shown in Figure 4C. Representative hit food samples were incubated with Aβ42 at 37 °C for 24 h, followed by negative staining with phosphotungstic acid. Scale bars, 500 nm.

Table S3. MSHTS activity of follow-up Camembert samples used in the cell viability assay.

Sample	Relationship to primary screen	MSHTS EC_{50}	Used in MTT
Primary Camembert hit	Primary automated MSHTS	0.064 ± 0.006 mg/mL	No
Camembert A	Follow-up sample	0.392 ± 0.185 mg/mL	Yes
Camembert B	Follow-up sample	0.203 ± 0.058 mg/mL	Yes

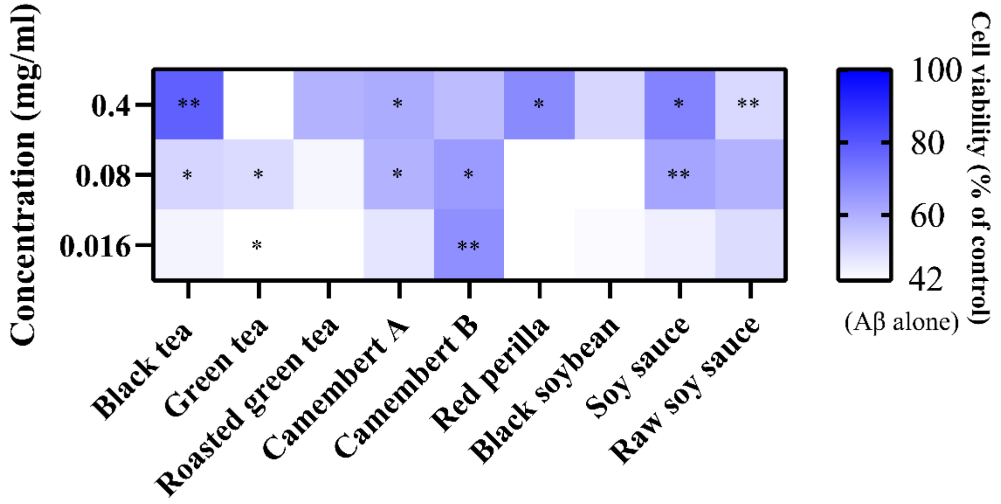

Figure S3. Full MTT assay results for selected hit and follow-up food samples.

Differentiated PC12 cells were treated with Aβ42 in the presence or absence of food samples, and cell viability was evaluated by MTT assay. Values were normalized to the DMSO-only control and are shown as mean ± SD. Asterisks indicate significant differences versus the Aβ42-alone group, calculated using two-sided Welch's *t*-test: $p < 0.05$, $p < 0.01$, and $p < 0.001$ for *, **, and ***, respectively. Camembert A and B refer to follow-up Camembert cheese samples distinct from the Camembert sample identified in the primary automated MSHTS.

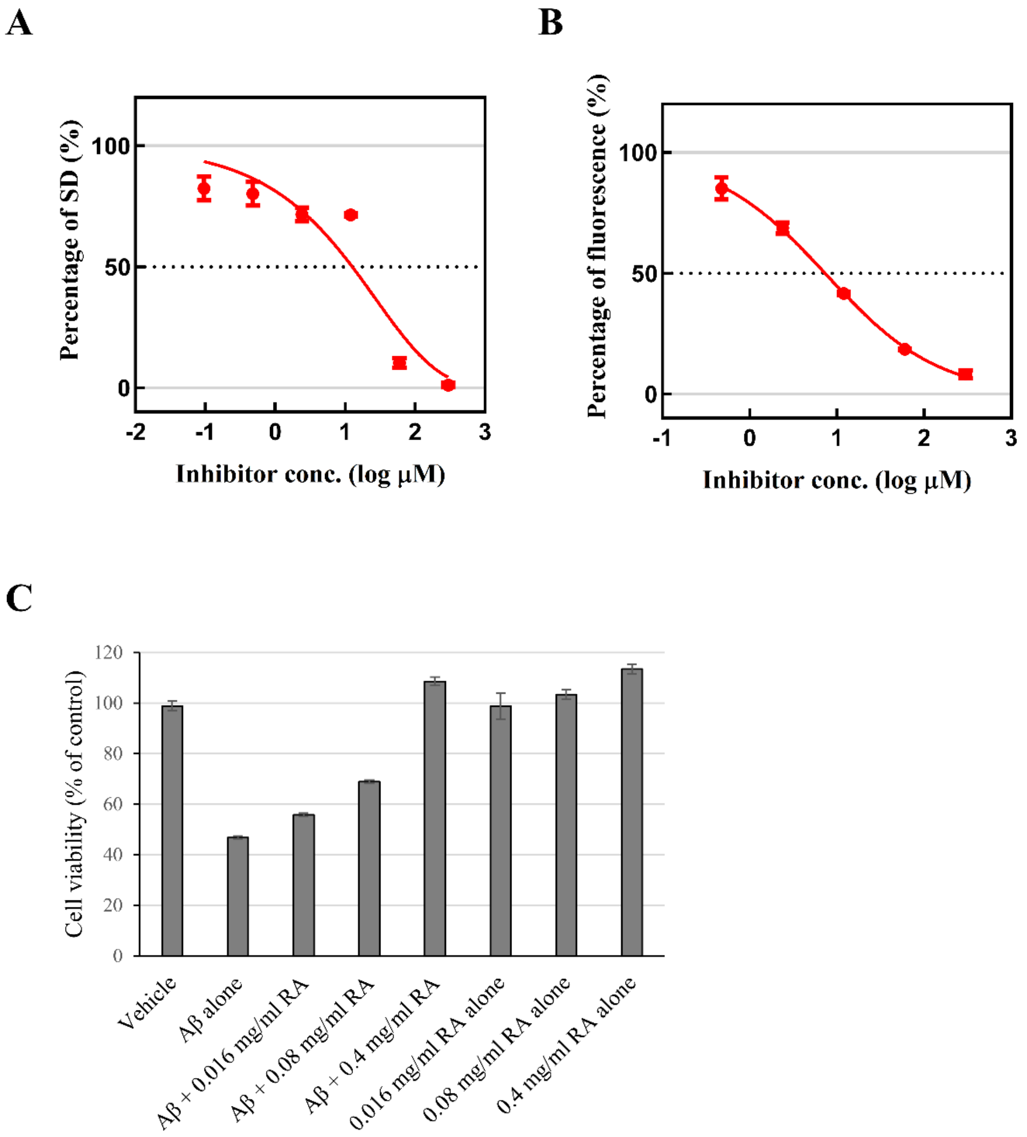

Figure S4. Validation of MSHTS, ThT, and MTT assays using rosmarinic acid.

(A) Concentration-dependent reduction in Aβ42 aggregation-related SD values by rosmarinic acid in the MSHTS assay. SD values were normalized to the Aβ42-alone condition and expressed as percentages. The estimated EC_{50} was 13.62 ± 1.25 μM. (B) Concentration-dependent reduction in Aβ42 aggregation-related ThT fluorescence by rosmarinic acid. Fluorescence values were normalized to the Aβ42-alone condition and expressed as percentages. The estimated EC_{50} was 7.25 ± 0.74 μM. (C) Effects of rosmarinic acid on Aβ42-induced reduction in cell viability estimated by the MTT assay. Absorbance values were corrected by subtracting sample-matched background signals for each rosmarinic acid concentration. Rosmarinic acid-alone controls were included to evaluate the

effect of rosmarinic acid itself on the MTT readout. Data are shown as mean ± SD.

Table S4. Statistical analysis of MTT assay results. Each treatment group was compared with the Aβ42-alone group using a two-sided Welch's *t*-test. Data were obtained from three replicate wells per group. Exact *p*-values are shown.

Sample	Concentration (mg/ml)	*p*-value
Black tea	0.4	0.00362
Black tea	0.08	0.01502
Black tea	0.016	0.26823
Green tea	0.4	0.07287
Green tea	0.08	0.31705
Green tea	0.016	0.03501
Roasted green tea	0.4	0.07287
Roasted green tea	0.08	0.31705
Roasted green tea	0.016	0.08189
Camembert A	0.4	0.03776
Camembert A	0.08	0.01536
Camembert A	0.016	0.57185
Camembert B	0.4	0.13663
Camembert B	0.08	0.00286
Camembert B	0.016	0.00910
Red perilla	0.4	0.03203
Red perilla	0.08	0.79776
Red perilla	0.016	0.77308
Black soybean	0.4	0.36491
Black soybean	0.08	0.93441
Black soybean	0.016	0.93398
Raw soy sauce	0.4	0.00936
Raw soy sauce	0.08	0.07112
Raw soy sauce	0.016	0.37289
Soy sauce	0.4	0.04849
Soy sauce	0.08	0.00737
Soy sauce	0.016	0.67201

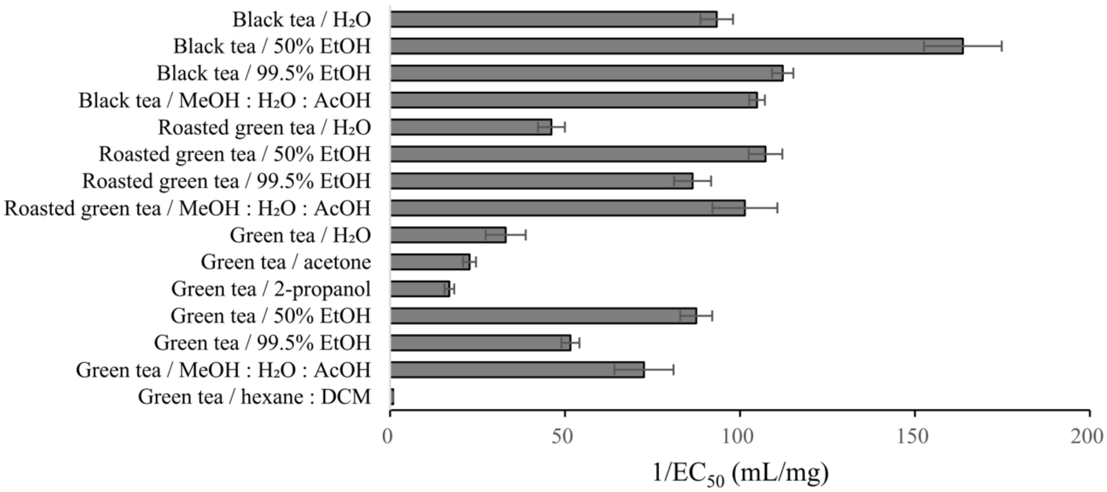

Figure S5. Preliminary comparison of extraction solvents using tea-related samples.

Green tea, roasted green tea, and black tea were extracted using the indicated solvent conditions, and Aβ42 aggregation-inhibitory activity was evaluated by MSHTS. The tested extraction solvents were hexane/DCM (1:1), MeOH/H_2O/AcOH (90:9.5:0.5), 99.5% EtOH, 50% EtOH, 2-propanol, acetone, and H_2O. Activity is shown as $1/EC_{50}$, with higher values indicating stronger Aβ42 aggregation-inhibitory activity. Data are presented as mean ± SD. H_2O, distilled water; EtOH, ethanol; MeOH, methanol; AcOH, acetic acid; DCM, dichloromethane.
